# Comparing Health Survey Data Cost and Quality Between Amazon’s Mechanical Turk and Ipsos’ KnowledgePanel: Observational Study

**DOI:** 10.2196/63032

**Published:** 2024-11-29

**Authors:** Patricia M Herman, Mary E Slaughter, Nabeel Qureshi, Tarek Azzam, David Cella, Ian D Coulter, Graham DiGuiseppi, Maria Orlando Edelen, Arie Kapteyn, Anthony Rodriguez, Max Rubinstein, Ron D Hays

**Affiliations:** 1 RAND Santa Monica, CA United States; 2 Department of Education University of California Santa Barbara Santa Barbara, CA United States; 3 Department of Medical Social Sciences Northwestern University Feinberg School of Medicine Chicago, IL United States; 4 RAND Pittsburgh, PA United States; 5 Brigham and Women’s Hospital Boston, MA United States; 6 RAND Boston, MA United States; 7 Center for Economic and Social Research Dana and David Dornsife School of Letters, Arts and Sciences University of Southern California Los Angeles, CA United States; 8 Division of General Internal Medicine and Health Services Research Department of Medicine University of California Los Angeles Los Angeles, CA United States

**Keywords:** data collection, probability panel, convenience sample, data quality, weighting, back pain, misrepresentation, Amazon, Mechanical Turk, MTurk, convenience panel, KnowledgePanel

## Abstract

**Background:**

Researchers have many options for web-based survey data collection, ranging from access to curated probability-based panels, where individuals are selectively invited to join based on their membership in a representative population, to convenience panels, which are open for anyone to join. The mix of respondents available also varies greatly regarding representation of a population of interest and in motivation to provide thoughtful and accurate responses. Despite the additional dataset-building labor required of the researcher, convenience panels are much less expensive than probability-based panels. However, it is important to understand what may be given up regarding data quality for those cost savings.

**Objective:**

This study examined the relative costs and data quality of fielding equivalent surveys on Amazon’s Mechanical Turk (MTurk), a convenience panel, and KnowledgePanel, a nationally representative probability-based panel.

**Methods:**

We administered the same survey measures to MTurk (in 2021) and KnowledgePanel (in 2022) members. We applied several recommended quality assurance steps to enhance the data quality achieved using MTurk. Ipsos, the owner of KnowledgePanel, followed their usual (industry standard) protocols. The survey was designed to support psychometric analyses and included >60 items from the Patient-Reported Outcomes Measurement Information System (PROMIS), demographics, and a list of health conditions. We used 2 fake conditions (“syndomitis” and “chekalism”) to identify those more likely to be honest respondents. We examined the quality of each platform’s data using several recommended metrics (eg, consistency, reliability, representativeness, missing data, and correlations) including and excluding those respondents who had endorsed a fake condition and examined the impact of weighting on representativeness.

**Results:**

We found that prescreening in the MTurk sample (removing those who endorsed a fake health condition) improved data quality but KnowledgePanel data quality generally remained superior. While MTurk’s unweighted point estimates for demographics exhibited the usual mismatch with national averages (younger, better educated, and lower income), weighted MTurk data matched national estimates. KnowledgePanel’s point estimates better matched national benchmarks even before poststratification weighting. Correlations between PROMIS measures and age and income were similar in MTurk and KnowledgePanel; the mean absolute value of the difference between each platform’s 137 correlations was 0.06, and 92% were <0.15. However, correlations between PROMIS measures and educational level were dramatically different; the mean absolute value of the difference across these 17 correlation pairs was 0.15, the largest difference was 0.29, and the direction of more than half of these relationships in the MTurk sample was the opposite from that expected from theory. Therefore, caution is needed if using MTurk for studies where educational level is a key variable.

**Conclusions:**

The data quality of our MTurk sample was often inferior to that of the KnowledgePanel sample but possibly not so much as to negate the benefits of its cost savings for some uses.

**International Registered Report Identifier (IRRID):**

RR2-10.1186/s12891-020-03696-2

## Introduction

### Background

Web-based panel data collection may offer cost, timing, and data quality benefits over traditional modes such as mail and telephone surveys [1,2]. However, these benefits depend on the web-based platform one uses, its available respondents, and the quality of the data obtained. These platforms range from access to curated probability-based panels, where individuals are selectively invited to join based on their membership in a representative population, to convenience panels, which are open for anyone to join [3]. The mix of respondents available on each platform can vary greatly in terms of representation of the population that is the focus of the investigation and in motivation to provide thoughtful and accurate responses.

Despite the additional labor required of the researcher to achieve a dataset ready for analysis, convenience panels are much less expensive to use than probability-based panels. However, it is important to understand what may be given up in terms of data quality for those cost savings. Researchers want to collect survey data that are representative, reliable, and valid [4]. They want to be sure that the responses gained are reasonable measures of the topic of interest (construct validity) [5]. In addition, they may want to generalize from the sample to a target population (external validity) [6]. Important questions for survey researchers are whether the likely cost saving from using a convenience panel is worth the potentially lower quality of the data obtained and under what conditions is that trade-off appealing.

Although respondents from all sources may provide distracted responses [7], convenience panels can attract respondents who use the platform as a main source of income [8-10]. This creates incentives both for careless or inattentive responses due to the desire to complete a survey quickly and for misrepresentation when respondents make false claims to qualify for a study [8,11]. Although careless or inattentive responses can both attenuate and increase expected correlations and can affect estimated factor structures [12], fraudulent or dishonest responses can pose an even greater threat to a study’s integrity by introducing systematic bias [5,8,13].

Convenience panels are also by design made up of individuals who are self-motivated to participate, and these individuals may not constitute a representative sample of the targeted underlying population [8]. A key technical requirement for standard statistics (eg, CIs) is that all members of the population of interest have a known, nonzero probability of being assigned to a survey [3,14,15]. This requirement is not met with a convenience sample and is of most concern when precise point estimates of population values are required [16,17]. Representativeness may also be important for unbiased estimates of relationships between variables of interest [16]. However, there are also study types (eg, explorative, methodological, and psychometric research) that may not require representative samples and benefit more from diversity [16].

### Objectives

In this study, we compared the cost of and data gathered using one of the most well-known and widely used convenience panels (Amazon’s Mechanical Turk [MTurk]) [18,19] to the cost of and data gathered using a nearly identical survey with a high-quality probability-based panel (KnowledgePanel) [17]. Given the different motivations and incentives facing each panel’s members, we first compared the quality of the data collected from MTurk and KnowledgePanel participants in terms of various measures of self-report accuracy, including whether additional data cleaning, especially in the MTurk sample, could improve data quality. Then, given any differences observed between the platforms in terms of sample demographic composition, we examined whether weighting could adequately address any differences observed and improve point estimates. Finally, we examined whether relationships between variables were similar across platforms and ended with a discussion of the situations under which a researcher would want to use each platform for data collection.

## Methods

To evaluate the advantages and disadvantages of using a convenience panel for web-based data collection versus using a probability-based panel, we fielded essentially the same survey using MTurk (August 31, 2021, to November 2, 2021) and then using KnowledgePanel (September 22, 2022, to October 2, 2022). We report our findings following both the Strengthening the Reporting of Observational Studies in Epidemiology statement [20] and the Checklist for Reporting Results of Internet E-Surveys [21].

### Data Sources

In this study, we used Amazon’s MTurk as an example of a well-known, inexpensive, fast, easily accessible convenience panel that can be used for data collection. As we note later, although data collection methods on MTurk have improved since, we used the methods recommended at the start of our data collection (August 2021). Therefore, hereafter, what we have labeled as MTurk data should be considered to represent a somewhat lower quality than what is possible now. MTurk was launched in 2005 [18,19,22]. Anyone who is aged ≥18 years and has a computing device connected to the internet is eligible to become an MTurk worker by creating a worker account. Once they have an account, they can select and complete any of the human intelligence tasks (HITs) available to them. Researchers can request a variety of HIT types from workers, such as identifying photo images, transcription, and responding to surveys [23]. Workers search for HITs using a search interface that shows them which ones they qualify for, the title of the HIT, the requester-generated description of the HIT, and the payment rate [10,24]. Workers are paid after successfully completing an HIT; the recommended pay rate is the federal minimum wage, although many HITs pay an even lower rate [6,25,26]. MTurk is an international panel with an estimated 226,500 workers in the United States, of whom 81,000 to 86,000 completed at least one HIT in 2016 to 2018 [27]. Because 25% to 42% of workers use MTurk as a main source of income [6,9,10] (see the examples on the website Finance Over Fifty [28] and Gigworker [29]), and to prevent automated methods to complete HITs, there are limits placed on the number of HITs to which a worker can respond per day [30].

KnowledgePanel [31] is a high-quality [17] probability-based panel founded in 1999 by Knowledge Networks and now owned by Ipsos Public Affairs [16,32]. Its >55,000 members are recruited using an address-based sampling methodology that uses the latest delivery sequence file of the US Postal Service. This probability-based sampling methodology improves population coverage, particularly for hard-to-reach individuals such as young adults and minority subgroups [31]. Most KnowledgePanel members have their own internet access and computers, but those who do not (approximately 5% [17]) are provided with a device and access as needed to ensure that the panel is representative of all adults in the United States regardless of phone, electronic device, and internet access status. Surveys are assigned to a random sample of panel members who meet desired sample criteria. Once assigned to a survey, the panel member receives an email notification, and reminders for response are sent via email followed by phone calls as needed. In general, panelists respond to an average of 2 to 3 surveys per month and receive a modest incentive through a point system (eg, 5000 points are worth approximately US $5) for each survey they screen in for and complete. Panelists do not receive payments for the screening surveys themselves; instead, those who do not screen in are entered into a sweepstakes. Panelists are also paid for maintaining their panel status (approximately US $4 to US $6 per month). Another study that used KnowledgePanel to gather health-related data found that the opportunity to learn health information about oneself was a strong motivation to participate [33]. Over 80% of KnowledgePanel participants are not a part of any other survey panel, and new panel members are recruited throughout the year to make up for attrition [34].

### Ethical Considerations

This study was approved by the RAND Human Subject Protection Committee (approval number 2019-0651-AM02). Respondents on each platform first faced a consent screen that laid out the study’s purpose; that their participation was voluntary, they could choose not to answer any question, and they could stop at any time; and that, responses would remain anonymous, only be used for research purposes, and only be reported for groups. Respondents gave consent by clicking to join the survey. The identities of the KnowledgePanel respondents were only known to Ipsos, and we had no access to MTurk respondents’ identities. The respondents to both surveys were compensated according to the usual procedures and amounts used for each platform.

### Comparison of Data Sources

Researcher access to platforms with probability-based panels requires a contract with the organization that owns the panel. Ipsos formatted and programmed our survey instrument, pretested it and then fielded it to 1 adult each from a nationally representative sample of households with email reminders sent every 3 days, delivered a fully formatted dataset containing the survey data with variable and value labels, created poststratification (nonresponse) statistical weights, and provided KnowledgePanel respondents’ standard demographic profile variables (ie, demographic data that Ipsos has on file for each panel member).

In contrast, a convenience panel such as MTurk is more of a self-service platform [9,18]. Access to MTurk workers is available to anyone with an Amazon Web Services account for the cost of the incentives paid to workers and a fee to Amazon. Requesters (researchers) must develop and format the survey in some generally accessible program (we used SelectSurvey [ClassApps, Inc]) and post it (much like a job advertisement) on the MTurk site available to workers looking for HITs, and they are responsible for releasing and monitoring the survey, as well as for data download, cleaning, and weighting.

We applied several quality assurance steps to enhance the data quality achieved using the MTurk platform [6,8,22,35-37]. Ipsos, the owner of KnowledgePanel, followed their usual (industry standard) protocols in fielding the survey. Table 1 provides a comparison of how the survey was fielded on each platform.

**Table 1:** Characteristics of Mechanical Turk (MTurk) and KnowledgePanel and the fielding of the survey on each platform.

Characteristic		MTurk	KnowledgePanel
**Background on each data collection platform**			
	Requirements for joining the panel	Platform is open to anyone aged ≥18 years with a computing device connected to the internet	Panelists must be invited to join based on a residential address sample from the US Postal Service; Ipsos provides computer or internet service if needed
	Financial agreement	Workers are paid based on the HITs^a^ they complete	Panelists receive points redeemable for cash per survey they screen in for and complete; entry into a sweepstakes, but no payment, is offered for the screening
	Motivation	Many use MTurk for income	Panelists do not earn enough to make income a motivation
**Fielding of this survey**			
	Respondent requirements	95% approval rate and ≥500 HITs; unique US IP address required^b^	Surveys are assigned to a random sample of panelists
	Monitoring respondent forums	Monitor worker forums for mention of the survey	N/A^c^
	Survey timing—how it is released to respondents	Microbatches of 9 surveys each released every hour until the sample size is achieved	Released to all assigned panelists at once
	Survey timing— time spent in the field for the baseline survey	August 31, 2021, to November 2, 2021 (63 days)	September 22, 2022, to October 2, 2022 (10 days)
	Survey timing—time limits on responses	After someone starts the survey, they must complete it within 48 hours, or they will be dropped	Survey is available for panelists to complete for up to 10 days
	Incentives for this survey	US $1.50 for general health survey plus US $2.00 for back pain survey	Respondents to the general health survey were entered into a monthly sweepstakes for prizes; those who qualified for and completed the back pain survey received 5000 points (approximately US $5)
**Survey characteristics**			
	Language	English only	English only
	Total number of items in the general health survey	101	100
	Differences in items between surveys	Included 8 additional demographic items that were available as profile variables from KnowledgePanel	Added EQ-5D-5L (n=6) and PROMIS^d^ social isolation (n=4) items
	Fake conditions (n=2)	Yes	Yes
	Advancing through the survey	1 item per page; required to click “Next” to advance	Sets of items with identical response categories were presented on 1 page in an “accordion” format, with each item’s response categories becoming visible as a response was given for the previous item; all other items were offered 1 per page, with clicking on “Next” required to advance
	Review step	No review step, but workers could use a back button to revise their answers	No review step, but respondents could use a back button to revise their answers
**Impact on researcher workload**			
	IRB^e^ implications—respondent identifiers	All anonymous but can be contacted for follow-up	Identity only known to Ipsos
	Formatting and programming the survey	Researcher builds it	Ipsos builds it with researcher input
	Survey initiation	Under researcher control	Ipsos schedules it with researcher input
	Data cleaning and quality control	Entirely the responsibility of the researcher; requires a higher level of effort	Ipsos does some quality control and cleaning, so there is a lower level of effort for the researcher

^a^HIT: human intelligence task.

^b^These qualifications have been recommended by a number of studies [6,8,22,36,37].

^c^N/A: not applicable.

^d^PROMIS: Patient-Reported Outcomes Measurement Information System.

^e^IRB: institutional review board.

### Survey Design

The survey was designed to enable psychometric evaluation and estimation of links and crosswalks between commonly used patient-reported outcome measures [38]. The first part of the survey was fielded as a survey of general health to all respondents [13,25,26] (ie, made available to all US-based MTurk workers and assigned to a nationally representative random sample of KnowledgePanel members). This portion of the survey contained >60 items from the Patient-Reported Outcomes Measurement Information System (PROMIS), demographics, and a list of health conditions that the respondent endorsed as “ever been told by a doctor or other health professional that” they had (14 items) or that they “currently” had (10 items). The second part of the survey was only offered to those who indicated current back pain and contained several established instruments used to measure back pain impact. The survey was fielded in English on both data collection platforms. Those who qualified for and completed the back pain survey at baseline were asked to complete that survey again at 3 and 6 months on the MTurk platform and were assigned that survey again at 6 months on KnowledgePanel.

Although early studies using MTurk found the platform to produce data of a quality equal to or better than those produced by many other sources and across several types of data [8,18], there have also been concerns about data quality [22,39]. To obtain the best quality data possible from MTurk, we followed several recommendations from the literature at that time. We limited the MTurk workers eligible for the survey to those who were located in the United States and had a good reputation [6,8,9,13,22,36,37] (ie, they had completed >500 HITs with a >95% approved-for-payment rating). MTurk workers are approved by their “employers” (researchers or requesters) using any criteria that the employers set up. In this study, workers were approved for payment if they reached the end of the survey and submitted the survey code available there. To ensure response consistency, we also eliminated from the sample anyone who completed the survey in an unrealistically short time [8,12,18,39]—less than 1 second per item—and we eliminated from the analytic file those who did not complete at least half of their assigned study items.

### Survey Implementation

As HITs are available to MTurk workers on a first-come-first-served basis, we limited any time-of-day or day-of-the-week bias by using microbatching—automatically releasing 9 surveys per hour, 24 hours per day, until our target sample was achieved (ie, over 2 months) [40]. An application called CloudResearch (formerly TurkPrime; Prime Research Solutions LLC) was used to accomplish this microbatching [41]. CloudResearch connects with the MTurk programming interface, enabling greater control over the survey process. CloudResearch has a tool that breaks a larger survey into microbatches of <10 participants each, ensuring sampling from individuals who are online at different times throughout the day and different days throughout the week. Releasing <10 surveys at a time also reduces the Amazon fee by 50%. In addition, CloudResearch allows for exclusion of people who have already completed the study (to prevent duplicate submissions) and enables anonymous emails to workers (allowing longitudinal data to be collected).

Some MTurk workers misrepresent their health status to increase their chances of being chosen for a survey [5,11,25,26,39]. Therefore, we used a recommended screening step to identify desired respondents for the back pain survey [5,7,25,26]. All respondents received a survey of general health that contained a list of health conditions that they indicated they had “ever” or “currently” had. This list included current back pain, but no indication was given that this was our target condition for the follow-up survey. Our pilot study work revealed that a substantial number of MTurk respondents (20%) endorsed having all or essentially all conditions listed—raising the question of whether some of these were fraudulent responses [5]. To identify those more likely to be honest respondents, we embedded 2 fake conditions in the list (“syndomitis” and “chekalism”) [8,13,42]. Those who endorsed either of these conditions were not offered the back pain survey even if they indicated that they had back pain. We used the same procedure to identify patients with back pain on the KnowledgePanel platform. For the MTurk platform, we also monitored online worker forums (eg, [43-46]) [26] to see whether there was any chatter about our survey and our fake conditions but found none.

The full text of all survey instruments fielded on each platform can be found on the Inter-university Consortium for Political and Social Research data repository (OPENICPSR-198049) [47].

### Analyses

The analyses used the data from the survey of general health. To create an analytic file for each data collection platform, we cleaned the data (eg, checking for out-of-range variables) and removed data from respondents whose response times were <1 second per item or who answered less than half of the items and created derived variables. Completion rates were calculated for each analytic sample, and the expenses and labor required for data collection on each platform were recorded. We then identified the respondents who endorsed one or both fake conditions and examined the quality of each platform’s data using several recommended metrics (eg, evidence of straight-lining and reliability; see the following paragraphs) including and excluding those respondents.

We used the κ statistic to assess the consistency of responses to 5 item pairs that addressed similar topics and had identical response categories [42,48] (refer to Multimedia Appendix 1 for the item pairs compared). Landis and Koch [49] provided a rule of thumb for the interpretation of κ—values of <0 indicated poor agreement, values of 0.00 to 0.20 indicated slight agreement, values of 0.21 to 0.40 indicated fair agreement, values of 0.41 to 0.60 indicated moderate agreement, values of 0.61 to 0.80 indicated substantial agreement, and values of 0.81 to 1.00 indicated almost perfect agreement. We also evaluated cases in which responses were too consistent [8,23,50] (ie, “straight-lining,” where respondents gave identical responses to consecutive sets of items [9,51]). We calculated the number and percentage of respondents who (1) chose the same response category for all sets of consecutive, same-response-category items; (2) chose the same response category for a set of 6 items on physical function where it would be unlikely that identical answers would make sense (ie, identical responses of “without any difficulty” [ratings of 5] were allowed, but identical responses of other response categories were not); and (3) chose the same response category for all items in one or both sets of 3 items on sleep where one item in each set was asked in a positive way and 2 were asked in a negative way (ratings of 3 [*somewhat* or *sometimes*] on a scale from 1 to 5 were allowed). We also calculated mean root of pairs [51] for each of these 3-item sleep sets—numbers closer to 1.0 indicated more straight-lining. Finally, we compared internal consistency reliability (Cronbach α [52]) for the 7 domain scales on the 29-item PROMIS (PROMIS-29) profile (physical function, fatigue, pain interference, depressive symptoms, anxiety, ability to participate, and sleep disturbance) plus the 2-item cognitive function scale using a *χ*^2^ test for independent samples [9,39,53-55].

Institutional review boards often require that respondents be allowed to skip or not answer any item in the survey. In this study, MTurk workers were only required to get to the end of the survey and submit a completion code to be approved and paid. KnowledgePanel panelists agreed upon joining the panel to answer all survey questions truthfully unless they felt uncomfortable doing so. Nevertheless, respondents on both platforms could leave many items unanswered, and the amount of missing data could affect conclusion validity and the generalizability of the study findings. Our data cleaning procedures removed those missing responses to more than half of the items from the analytic sample, and then we calculated the proportion of respondents in that sample for each platform who completed all items in the survey of general health. Finally, we reported the response burden for each platform in terms of the Winsorized average and median and ranges of time it took to complete the survey of general health, in each case after capping the top 2.5% of durations to the 97.5th percentile value [56].

All the aforementioned data quality checks were performed on the full sample for the survey of general health. However, that section of the survey did not include any open-ended questions where free-text responses could be examined [23,39]. The back pain survey (only offered to those who endorsed current back pain and did not endorse a fake condition) included an open-ended question—“What does chronic pain mean to you?” Therefore, we compared responses from MTurk and KnowledgePanel in terms of whether (1) the response was nonsensical (not related to the question, eg, “good” or “text”) and (2) the response was copied from a common source (ie, contained a string of at least 10 words that made up at least 75% of the response and were identical to those found in one or more other open-ended responses). We also reported the number of those who completed the back pain survey who went on to complete the 3- and 6-month follow-up surveys on MTurk and the 6-month follow-up survey on KnowledgePanel.

We examined the representativeness of the MTurk and KnowledgePanel samples by comparing the point estimates from each to US national estimates first including and then excluding those who endorsed one or both fake conditions and again after applying weights to those who did not endorse a fake condition [57]. Estimates for the demographic variables that were used for weighting (age, gender, race and ethnicity, income, educational level, and region), the PROMIS-29 scales [58] and physical and mental health summary scores [59], and disease prevalence were compared.

The KnowledgePanel sample was selected to match the population of adults in the United States using a probability-proportional-to-size procedure that used a set of design weights as measures of size. Therefore, even before any additional weighting, we would expect that sample to be closer to national estimates (March 2022 supplement of the Current Population Survey) than the MTurk sample. After the KnowledgePanel survey data were collected, these design weights were adjusted using poststratification weights, which can help account for any differential nonresponse [34]. Ipsos used an iterative proportional fitting (raking) procedure with trimming and scaling to produce the final weights. For MTurk, we followed a similar procedure and created weights to account for both the original composition of respondents and for any nonresponse and examined how useful weighting was in improving the accuracy of the point estimates from each platform [57,60].

Finally, some researchers are more interested in relationships between variables and argue that results of multivariate analyses are more similar between convenience samples and probability-based samples than those of univariate analyses [14,57,61-63]. Correlations measure the strength and direction of bivariate relationships. We provided a correlation matrix (using unweighted data excluding those who endorsed a fake condition and including 95% CIs) to compare platforms in terms of the linear relationships between the variables measured in the survey of general health—the 7 PROMIS-29 domain scale T-scores [58], the mental and physical health summary T-scores both from the PROMIS-29 [59] and from the PROMIS Global Health items [64], the Impact Stratification Score [65], age, educational level, and income. The coefficients for each dataset are shown above and below each other in the matrix to allow for “ocular” comparisons. Although we provide some estimates of the differences observed (eg, means and maximum absolute differences), we leave it to the reader to judge the extent to which these coefficients are similar enough for their analytic needs.

## Results

### Comparison of Labor Days and Dollar Expenditures Required for Use of Each Platform

Table 2 shows the study team labor days and dollar expenditures involved in each step of generating the data and analytic datasets gathered from each platform for this study. Although the labor needs for the study team were lower, the dollar expenditures to obtain these data from KnowledgePanel were substantially (approximately 9 times) higher. Of course, gathering smaller samples would have cost less, but the relative size of the expenses would increase because, although the MTurk expenses were solely determined by sample size, the KnowledgePanel expenses included components relatively insensitive to sample size.

**Table 2:** Labor and expenditures to create the final analytic datasets from each platform.

	Mechanical Turk		KnowledgePanel	
	Labor days, n	Expenditures (US $)	Labor days, n	Expenditures (US $)
Formatting and programming of survey instrument	3.3	0	0	236,178^a^
Pretests and then full fielding of the survey	2.8	0	0	236,178^a^
Incentive payments	1.7	27,146	0	236,178^a^
Fully formatted SAS^b^ dataset with appropriate variable and value labels	1.7	0	0	236,178^a^
Weighting	1.5	0	0	236,178^a^
Creation of final analytic dataset	15.8	0	7.8	0
Total	26.7	27,146	7.8	236,178^a^

^a^These tasks did not individually cost US $236,178. This amount is the total across all these tasks.

^b^SAS: Statistical Analysis System.

### Comparison of the Numbers of Respondents and Quality of the Data From Each Platform

Tables 3 and 4 presents the comparison of various measures of data quality between platforms. Neither platform had any respondents with response times of <1 second per item. Analytic datasets were created by removing those who did not answer at least half of the items they were assigned (designated as incompletes); the percentage of incomplete surveys removed from the MTurk dataset was almost 9 times that of incomplete surveys removed from the KnowledgePanel dataset. Our completion rates based on the analytic datasets were 49.6% (6750/13,608) for MTurk (with 13,608 being the number of surveys available [63 days × 24 hours × 9 surveys released per hour]) and 57.2% (4134/7224) for KnowledgePanel (with 7224 being the number of panelists assigned to the survey).

**Table 3:** Numbers of respondents and completion rates for each platform.

	Mechanical Turk	KnowledgePanel
Surveys fielded, n	13,608	7224
Final total upon field close, n	6997	4149
Surveys with too short response times (<1 second per item), n (%)	0 (0)	0 (0)
Incomplete surveys (missing more than half of the items), n/N (%)	247/6997 (3.5)	15/4149 (0.4)
Analytic dataset, n	6750	4134
Completion rate, n/N (%)	6750/13,608 (50)	4134/7224 (57)
Participants who endorsed fake conditions, n/N (%)	975/6750 (14.4)	19/4134 (0.5)

**Table 4:** Indicators of quality for the survey data collected on each platform^a^.

			All (n=6750)			No fake conditions (n=5775)		All (n=4134)		No fake conditions (n=4115)	
**Response consistency, κ (quadratically weighted for agreement; see Multimedia Appendix 1 for the item pairs)**											
	2 items on pain interference	0.83			0.83			0.91	0.91		
	2 items on trouble doing	0.72			0.73			0.77	0.77		
	2 items on problems with sleep	0.73			0.72			0.67	0.67		
	2 items on ability to concentrate or focus	0.46			0.47			0.71	0.71		
	2 items on memory	0.55			0.56			0.70	0.70		
**Measures of straight-lining**											
	Participants who straight-lined all sets of same-response-category items, n (%)	20 (0.3)			11 (0.2)			6 (0.1)	5 (0.1)		
	Participants who straight-lined the 6-item physical function set (no ratings of 5), n (%)	98 (1.5)			55 (1)^b^			34 (0.8)	34 (0.8)		
	Participants who straight-lined one or both sleep item sets (no ratings of 3), n (%)	611 (9.1)			441 (7.6)^c^			237 (5.7)	233 (5.7)^d^		
	Root of pairs (first sleep set; larger=more), mean (SD)^e^	0.43 (0.26)			0.41 (0.25)^d^			0.39 (0.25)	0.39 (0.24)^d^		
	Root of pairs (second sleep set; larger=more), mean (SD)^e^	0.45 (0.28)			0.43 (0.27)^d^			0.42 (0.26)	0.42 (0.26)		
**Internal consistency reliability**											
	Physical function, Cronbach α for PROMIS^f^ scales	0.881			0.894^d^			0.938	0.938^d^		
	Fatigue, Cronbach α for PROMIS scales	0.919			0.924^b^			0.942	0.943^d^		
	Pain interference, Cronbach α for PROMIS scales	0.940			0.938			0.963	0.963^d^		
	Depressive symptoms, Cronbach α for PROMIS scales	0.918			0.922			0.939	0.940^d^		
	Anxiety, Cronbach α for PROMIS scales	0.900			0.901			0.906	0.907		
	Ability to participate, Cronbach α for PROMIS scales	0.922			0.923			0.947	0.947^d^		
	Sleep disturbance, Cronbach α for PROMIS scales	0.775			0.840^j^			0.875	0.875^d^		
	Cognitive function (2 items), Cronbach α for PROMIS scales	0.759			0.770			0.844	0.844^d^		
	Cognitive function (5 items), Cronbach α for PROMIS scales	0.856			0.862			0.905	0.905^d^		
	Participants who completed all items in the general health survey, n (%)	6152 (91.1)			5379 (93.1)^d^			3345 (80.9)	3335 (81)^d^		
	Response burden (minutes)—general health survey, Winsorized mean (SD)	21 (14)			20 (13)			11 (7)	11 (7)		
	Response burden (minutes)—general health survey, median (range)	17 (3-55)			15 (3-55)^d^			10 (2-38)	10 (2-38)^d^		
**Only asked of back pain survey respondents**											
	Eligible for back pain survey (endorsed back pain but no fake conditions), n (%)	—^g^		2307 (39.9)^h^			—				1533 (37.3)^i^
	Responded to back pain survey (back pain analytic file), n (%)	—		1972 (34.1)^h^			—				1531 (37.2)^i^
	Response burden (minutes)—general health survey+back pain survey, Winsorized mean (SD)	—		32 (13)			—				25 (14)
	Response burden (minutes)—general health survey+back pain survey, median (range)	—		30 (5-58)			—				21 (3-75)^d^
	Nonsense responses to “what is chronic?” n (%)	—		32 (1.6)^j^			—				4 (0.3)^d,k^
	Copied or identical text in response to “what is chronic?” n (%)	—		212 (10.8)^j^			—				0 (0.0)^d,k^
	Responded to 3-month follow-up surveys, n (%)	—		1077 (54.6)^j^			—				—
	Responded to 6-month follow-up surveys, n (%)	—		845 (42.8)^j^			—				1256 (82.0)^k^

^a^Indicators of statistical strength of differences between Mechanical Turk—all and Mechanical Turk—no fake conditions are shown in the Mechanical Turk no fake conditions column and between Mechanical Turk—no fake conditions and KnowledgePanel—no fake conditions are shown in the KnowledgePanel no fake conditions column. We used 2-tailed *t* tests for comparisons of means and *χ*^2^ tests for comparisons of frequencies and for the comparisons of α coefficients.

^b^*P*<.05.

^c^*P*<.01.

^d^*P*<.001.

^e^Mean root of pairs=the mean of the root of the absolute differences between all pairs of items in a battery, rescaled to range from 0 (least straight-lining) to 1 (most straight-lining).

^f^PROMIS: Patient-Reported Outcomes Measurement Information System.

^g^Not applicable.

^h^n=5775.

^i^n=4115.

^j^n=1972.

^k^n=1531.

The rate of respondents endorsing one or both fake conditions was almost 30 times higher on the MTurk than the KnowledgePanel platform. As the incidence of fake condition endorsement was so low in the KnowledgePanel dataset, removing those who endorsed these conditions had little impact on its data quality. However, removing those who endorsed a fake condition from the MTurk analytic dataset generally improved its data quality, in some cases substantially [35]. Removing those who endorsed a fake condition had little impact on response consistency (ie, κ values between similar pairs of items barely increased) but did reduce the incidence of straight-lining (ie, respondents giving identical responses to consecutive sets of similar items); improve internal consistency reliability, especially for a 4-item scale with reverse-coded items (sleep disturbance) and for physical function; and increase the proportion of respondents who completed all items. On average, KnowledgePanel respondents took a little more than half the time that MTurk respondents took to complete the general health survey. However, despite this response speed, and even after removing those who endorsed the fake conditions from the MTurk dataset, the KnowledgePanel dataset showed better response consistency—for the concentration/focus and memory pairs, estimated κ values from KnowledgePanel were “substantial” versus “moderate” for MTurk. KnowledgePanel respondents exhibited less straight-lining, better internal consistency reliability, and fewer nonsense and copied responses for the open-ended back pain survey item and had a substantially (39 percentage points) higher completion rate for the 6-month follow-up survey. Nevertheless, significantly fewer KnowledgePanel respondents than MTurk respondents completed all items in the survey of general health.

### Comparison of the Characteristics of Respondents on Each Platform

Table 5 shows the demographic characteristics of respondents for the 2 platforms. The biggest impact of removing those who endorsed a fake condition in the MTurk sample was that the proportion of respondents who identified as Hispanic individuals dropped from 19.54% (1319/6750) to 14.06% (812/5775) in the sample; 52% (507/975) of those who endorsed a fake condition identified as Hispanic individuals. Before weighting and when comparing those who did not endorse a fake condition, MTurk respondents were younger, with most respondents in the age category of 30 to 44 years, whereas the age category with the most respondents in KnowledgePanel was ≥60 years, better matching national estimates. More MTurk respondents were male, fewer identified as non-Hispanic Black individuals, and more identified as Hispanic individuals than those from KnowledgePanel. While both platforms had similar proportions of respondents with a master’s degree or higher, KnowledgePanel respondents included a larger proportion of those with an educational level of high school or lower than MTurk (1369/4115, 33.27% vs 480/5775, 8.31%), and MTurk had a much larger proportion of respondents with a bachelor’s degree (2807/5775, 48.61% vs 907/4115, 22.04%). MTurk had more respondents in the income categories of <US $100,000 per year, and KnowledgePanel had more in the category of ≥US $100,000 per year (4984/5775, 86.3% vs 1740/4115, 42.28%). Similar proportions of MTurk and KnowledgePanel respondents resided in each of the 4 US census regions.

**Table 5:** Comparison of the demographic characteristics before and after weighting between the Mechanical Turk (MTurk) and KnowledgePanel samples and national estimates.

			MTurk						KnowledgePanel							National estimates (%)^a^
			All (n=6750)	No fake conditions (n=5775)		Weighted (n=5775)		All (n=4134)			No fake conditions (n=4115)		Weighted (n=4098)			
Age (years), mean (SD)			39 (12)	40 (12)		46 (15)		52 (18)			52 (18)		48 (18)		47.5	
**Age (years), n (%)**																
	18-29	1268 (18.8)		1130 (19.6)	1158 (20.1)		560 (13.5)			559 (13.6)		817 (19.9)		19.9		
	30-44	3514 (52.1)		2913 (50.4)	1484 (25.7)		951 (23)			945 (23)		1059 (25.8)		25.9		
	45-60	1392 (20.6)		1204 (20.8)	1349 (23.4)		914 (22.1)			909 (22.1)		980 (23.9)		24		
	≥60	499 (7.4)		466 (8.1)	1706 (29.5)		1709 (41.3)			1702 (41.4)		1242 (30.3)		30.3		
**Gender, n (%)**																
	Female	2931 (43.4)		2617 (45.3)	2882 (49.9)		2040 (49.3)			2033 (49.4)		2096 (51.1)		50.7		
	Male	3695 (54.7)		3047 (52.8)	2754 (47.7)		2055 (49.7)			2044 (49.7)		1962 (47.9)		49.3		
**Race and ethnicity, n (%)**																
	Hispanic	1319 (19.5)		812 (14.1)	979 (17)		499 (12.1)			495 (12)		702 (17.1)		17.2		
	Multiracial	124 (1.8)		121 (2.1)	80 (1.4)		136 (3.3)			136 (3.3)		57 (1.4)		1.4		
	Non-Hispanic Black	531 (7.9)		477 (8.3)	686 (11.9)		411 (9.9)			411 (10)		495 (12.1)		12		
	Non-Hispanic White	4362 (64.6)		3968 (68.7)	3537 (61.2)		2894 (70)			2880 (70)		2542 (62)		62		
	Non-Hispanic other	330 (4.9)		328 (5.7)	418 (7.2)		194 (4.7)			193 (4.7)		301 (7.3)		7.3		
**Educational attainment, n (%)**																
	No high school diploma or GED^b^	18 (0.3)		18 (0.3)	540 (9.4)		278 (6.7)			277 (6.7)		394 (9.6)		9.6		
	High school graduate or GED	466 (6.9)		462 (8)	1650 (28.6)		1100 (26.6)			1092 (26.5)		1192 (29.1)		29.2		
	Some college or associate’s degree	1405 (20.8)		1384 (24)	1495 (25.9)		1088 (26.3)			1083 (26.3)		1083 (26.4)		26.4		
	Bachelor’s degree	3380 (50.1)		2807 (48.6)	1269 (22)		909 (22)			907 (22)		784 (19.1)		34.8^c^		
	Master’s degree or higher	1385 (20.5)		1024 (17.7)	698 (12.1)		759 (18.4)			756 (18.4)		644 (15.7)		34.8^c^		
**Household income (US $), n (%)**																
	<10,000	275 (4.1)		249 (4.3)	302 (5.2)		121 (2.9)			121 (2.9)		144 (3.5)		3.6		
	10,000-49,999	2812 (41.7)		2438 (42.2)	1764 (30.5)		1007 (24.4)			999 (24.3)		1022 (24.9)		24.9		
	50,000-99,999	2821 (41.8)		2297 (39.8)	1597 (27.7)		1258 (30.4)			1255 (30.5)		1172 (28.6)		28.6		
	≥100,000	762 (11.3)		727 (12.6)	2037 (35.3)		1748 (42.3)			1740 (42.3)		1760 (42.9)		42.9		
**Region of the country, n (%)**																
	Northeast	1125 (16.7)		1012 (17.5)	992 (17.2)		751 (18.2)			745 (18.1)		710 (17.3)		17.4		
	Midwest	1292 (19.1)		1134 (19.6)	1176 (20.4)		900 (21.8)			896 (21.8)		845 (20.6)		20.6		
	South	2670 (39.6)		2283 (39.5)	2183 (37.8)		1522 (36.8)			1514 (36.8)		1568 (38.3)		38.3		
	West	1582 (23.4)		1281 (22.2)	1351 (23.4)		961 (23.2)			960 (23.3)		974 (23.8)		23.7		

^a^National estimates on this table are all from the Current Population Survey Annual Social and Economic Supplement (March 2022) for persons aged ≥18 years in the United States.

^b^GED: General Educational Development.

^c^This value reflects the combined sum of both rows.

Both samples were weighted using the demographic variables shown in Table 5 (age, gender, race and ethnicity, educational level, income, and region). Weighting these samples resulted in datasets that generally well matched the national estimates. The KnowledgePanel weights (maximum weight of 2.8) brought those data completely in line with national estimates, whereas weighting the MTurk data allowing for a maximum weight of 30 brought those data within a total absolute imbalance of 0.01 across the 6 demographic variables used to construct the weights.

Table 6 shows other characteristics of the samples from each platform. In the full dataset, the MTurk sample generally had worse PROMIS T-scores than the KnowledgePanel sample and national estimates for all scales. Removing respondents who endorsed a fake condition generally brought the average PROMIS T-score on each scale for the MTurk respondents closer to those of KnowledgePanel and to national estimates. Only considering PROMIS scores that differed by >2 T-scores (ie, a “small” effect size) compared to national estimates after weighting, the MTurk sample had more anxiety, better ability to participate in social roles and activities, and a better mental health summary score than national estimates. The KnowledgePanel sample had less fatigue and better ability to participate in social roles and activities, cognitive function, and mental and physical health summary scores than national estimates.

**Table 6:** Characteristics of the samples from each platform.

			Mechanical Turk						KnowledgePanel								National estimates
			All (n=6750)		No fake conditions (n=5775)		Weighted (n=5775)	All (n=4134)				No fake conditions (n=4115)		Weighted (n=4098)			
**PROMIS-29+2^a^ scale T-scores, mean (SD)**																	
	Physical function	48 (8)		49 (8)		50 (8)				51 (8)	51 (8)		51 (8)		50 (10)^b^		
	Pain interference	53 (10)		51 (9)		50 (9)				49 (9)	49 (9)		49 (9)		50 (10)^b^		
	Fatigue	51 (10)		50 (10)		49 (11)				48 (10)	48 (10)		48 (10)		50 (10)^b^		
	Depressive symptoms	54 (10)		53 (10)		51 (10)				48 (9)	48 (9)		49 (9)		50 (10)^b^		
	Anxiety	56 (10)		54 (10)		52 (10)				49 (9)	49 (9)		49 (9)		50 (10)^b^		
	Ability to participate	52 (10)		53 (9)		55 (10)				56 (9)	56 (9)		56 (9)		50 (10)^b^		
	Sleep disturbance	50 (9)		50 (9)		49 (10)				49 (9)	49 (9)		49 (9)		50 (10)^b^		
	Cognitive function	49 (9)		50 (9)		51 (9)				52 (9)	52 (9)		52 (9)		50 (10)^b^		
	Mental health summary score	48 (9)		50 (9)		52 (10)				53 (9)	53 (9)		53 (9)		50 (10)^b^		
	Physical health summary score	48 (9)		49 (9)		51 (9)				51 (9)	51 (9)		52 (9)		50 (10)^b^		
	Number of health conditions endorsed (out of 24 possible conditions)	6.0 (6.0)		3.8 (3.4)		3.7 (3.1)				4.0 (3.2)	4.0 (3.2)		3.8 (3.1)		—^c^		
**Disease prevalence, n (%)**																	
	Anxiety (ever)	2293 (33.97)		1618 (28.02)		1520 (26)				814 (19.69)	806 (19.59)		845 (21)		33.7^d^		
	Depression (ever)	2737 (40.55)		2005 (34.72)		1688 (29)				829 (20.05)	820 (19.93)		832 (20)		18.4^e^		
	Hypertension (ever)	2423 (35.9)		1578 (27.32)		1786 (31)				1581 (38.24)	1570 (38.15)		1381 (34)		47.3^f^		
	Asthma (ever)	1561 (23.13)		889 (15.39)		770 (13)				536 (12.97)	532 (12.93)		547 (13)		13.5^g^		
	Diabetes (ever)	1360 (20.15)		678 (11.74)		542 (9)				554 (13.4)	548 (13.32)		496 (12)		13^f^		
	Heart disease (ever had any of these: heart attack, CHD^h^, or angina—3 different)	1428 (21.16)		538 (9.32)		414 (7)				301 (7.28)	295 (7.17)		237 (6)		5.5^g^		
	COPD^i^ (ever)	923 (13.67)		293 (5.07)		369 (6)				193 (4.67)	191 (4.64)		165 (4)		5.6^j^		
	Cancer (ever)	948 (14.04)		295 (5.11)		459 (8)				421 (10.18)	417 (10.13)		325 (8)		7.5^g^		
	Stroke (ever)	921 (13.64)		254 (4.4)		133 (2)				109 (2.64)	106 (2.58)		88 (2)		2.8^g^		
	Neck pain (currently)	2077 (30.77)		1393 (24.12)		1136 (20)				816 (19.74)	811 (19.71)		781 (19)		15.7^k^		
	Back pain (currently)	3035 (44.96)		2307 (39.95)		2136 (37)				1541 (37.28)	1533 (37.25)		1467 (36)		39^g^		
	Responded to the back pain survey	—		1972 (34.15)		1872 (32)				—	1531 (37.21)		1467 (36)		—		
	Nonspecific back pain	—		1471 (74.59)^l^		1228 (66)				—	983 (64.21)^m^		981 (67)		—		
	Chronic (>3-month duration)	—		1539 (78.04)^l^		1593 (85)				—	1379 (90.07)^m^		1312 (90)		—		
	Chronic (RTF^n^ definition)	—		1174 (59.53)^l^		1169 (62)				—	868 (56.69)^m^		826 (57)		—		
	Chronic (provider identified)	—		495 (25.1)^l^		624 (33)				—	552 (36.05)^m^		498 (34)		—		
	Chronic (patient identified)	—		1156 (58.62)^l^		1245 (67)				—	865 (56.5)^m^		801 (55)		—		
	Chronic (any definition)	—		1719 (87.17)^l^		1718 (92)				—	1400 (91.44)^m^		1330 (91)		—		

^a^PROMIS-29+2: 29-item Patient-Reported Outcomes Measurement Information System profile plus 2 cognitive items.

^b^Patient-Reported Outcomes Measurement Information System T-scores have a mean of 50 and an SD of 10 in a national sample.

^c^There is no national estimate available for this characteristic.

^d^National Comorbidity Survey Replication.

^e^Centers for Disease Control and Prevention Morbidity and Mortality Weekly Report [66].

^f^National Health and Nutrition Examination Survey 2017 to 2018.

^g^2019 National Health Interview Survey data.

^h^CHD: coronary heart disease.

^i^COPD: chronic obstructive pulmonary disease.

^j^2017 Behavioral Risk Factor Surveillance System.

^k^2013 to 2015 National Health Interview Survey data.

^l^n=1972.

^m^n=1531.

^n^RTF: National Institutes of Health Pain Consortium’s 2012 research task force.

Removing MTurk respondents who endorsed a fake condition substantially reduced the total number of health conditions endorsed and the number endorsing each condition, and weighting these data further reduced the number claiming to have each condition except for hypertension and cancer. Removing those who endorsed a fake condition had little effect on the KnowledgePanel results, but weighting those data generally resulted in slightly lower condition prevalence, with the greatest reduction in the prevalence of hypertension. The weighted data showed that the MTurk respondents had more anxiety and depression and slightly more back and neck pain, whereas the KnowledgePanel respondents had more hypertension and diabetes. Both platforms underestimated anxiety and hypertension compared to available national estimates and overestimated depression and neck pain. After removing those who endorsed a fake condition, the prevalence of back pain was remarkably similar between the samples. However, MTurk respondents who were eligible for the back pain survey were asked whether they wanted to go on to take that survey, and 15% (335/2307) opted out, dropping back pain prevalence in that sample substantially below national averages. KnowledgePanel respondents did not receive that opt-out question. After weighting, the proportions with nonspecific back pain were similar between samples, but the prevalence of each type of chronic back pain differed. Compared to KnowledgePanel, the proportion of the MTurk sample who said that they thought their back pain is chronic was 12 percentage points higher.

### Comparison of Correlations Between Variables Measured on Each Platform

Finally, correlation coefficients estimated using the data from each platform showed many similarities but some important differences. In the correlation matrix using the full sample (Multimedia Appendix 2), the correlation coefficients for the PROMIS measure variables (ie, all but the last 3 rows) for each dataset were all in the same direction and tended to be similar in magnitude, with the KnowledgePanel correlation coefficient being larger 55% of the time. Of those 105 correlation pairs, the mean absolute value of the difference between each platform’s correlations was 0.05, with differences of >0.05 in 45% of the correlations, >0.10 in 24% of the correlations, and >0.15 in 7% of the correlations. Only one correlation pair (between the Impact Stratification Score and PROMIS global mental health summary score) had an absolute value difference of >0.20 (0.202). The MTurk sample’s correlation coefficients between age, income, and educational level and PROMIS measures were larger than those of KnowledgePanel 85% of the time. However, the differences among the coefficients for PROMIS measures, age, and income followed a similar pattern to that observed between the PROMIS measures alone. It is notable that the 17 correlation coefficient pairs between educational level and the other variables differed markedly by platform. More than half (9/17, 53%) of the correlation pairs had opposite signs (with the MTurk coefficient indicating a negative relationship between educational level and better health as measured using the PROMIS, which is contrary to theory [67]), and the absolute difference among 7 correlation coefficient pairs was >0.20, the average difference across all 17 pairs was 0.15, and the largest difference was 0.29.

## Discussion

### Principal Findings

#### Overview

KnowledgePanel and MTurk are examples of 2 types of web-based platforms that differ markedly in terms of the cost and investigator effort required for data collection but also in terms of the respondents and the quality of the data obtained [3]. KnowledgePanel members are experienced survey respondents, and new members are recruited to the platform as needed to maintain a nationally representative panel. Ipsos then randomly draws a sample from this panel and assigns them to a survey. In contrast, anyone aged ≥18 years with a computing device connected to the internet can become an MTurk worker, and when they want to, they can search for and respond to HITs such as a survey. On KnowledgePanel, a random representative sample is assigned to a survey; on MTurk and similar convenience opt-in panels, although one can take some steps to improve data quality, one generally takes what one can get. As expected, the quality of the data obtained from KnowledgePanel respondents was measurably better than that of the data obtained from MTurk, but the KnowledgePanel sample analyzed in this study cost thousands of dollars more. This study provides information with which to weigh the benefits of KnowledgePanel’s representative, assigned sample against the cost savings afforded by platforms such as MTurk.

There are three main considerations when comparing the data received from different sources: (1) accurate responses in terms of both potential misrepresentation and thoughtful/careful answers, (2) quality of point estimates, and (3) comparisons of multivariate analyses. In the following sections, we compare the results for our MTurk and KnowledgePanel samples (each gathered according to the criteria described in Table 1) with regard to these considerations.

#### Misrepresentation and Thoughtful/Careful Answers

In this study, our prescreening, including the use of fake conditions to identify and remove respondents who were misrepresenting themselves (or at least careless), helped improve data quality in the MTurk sample. MTurk respondents endorsed a fake health condition at almost 30 times the rate observed in KnowledgePanel (975/6750, 14.4% vs 19/4134, 0.5%). However, even after those who endorsed a fake condition were removed, the quality of the KnowledgePanel data remained somewhat superior. We also offered fake conditions in the follow-up surveys, and according to another study of these data, it is possible that almost a quarter of the MTurk sample were misrepresenting themselves or careless [35]—an estimate that is within the range of misrepresentation observed in other MTurk surveys [13]. Our longitudinal data made it possible to estimate the percentage of misrepresenters more accurately than a single administration. However, some misrepresenters likely remained unidentified. The comparison of the relative data quality of MTurk and KnowledgePanel could be different if we knew with certainty those who were misrepresenting themselves.

Removing respondents who endorsed a fake condition substantially reduced the number and prevalence of the health conditions endorsed by MTurk respondents, but it also reduced the number of respondents who identified as Hispanic individuals. It is unclear why more than half of those who endorsed a fake condition also identified as being Hispanic. It could be that this is a further instance of erroneous reporting, or it could be that respondents thought that claiming Hispanic ethnicity would increase their chances of being chosen for further surveys. In any case, a systematic review of 54 MTurk studies found that racial and ethnic minority groups were more likely to be excluded using a variety of screening criteria [7].

It is unclear why a larger proportion of MTurk respondents (247/6997, 3.5%) than of KnowledgePanel respondents (15/4149, 0.4%) were removed from the analytic dataset for answering less than half the questions but more remaining MTurk respondents (6152/6750, 90.1% and 5379/5775, 93.1% before and after excluding those who endorsed a fake condition, respectively) than KnowledgePanel respondents (3345/4134, 80.9% and 3335/4115, 81%) answered all the questions in the main survey. The consent screen for both sets of respondents included that their participation was voluntary, they could choose not to answer any question, and they could stop at any time. However, once started, the MTurk workers may have felt an implicit obligation to complete the task fully to receive a good rating.

One result that was surprising is that, compared to the MTurk respondents, the KnowledgePanel respondents provided higher-quality data according to several measures while being substantially faster—11 versus 20 minutes for those who only completed the survey of general health and 25 versus 32 minutes for both the general health and back pain surveys combined. There could be many reasons for this, including that KnowledgePanel respondents tend to be long-term, experienced members of a panel paid to respond to surveys or that MTurk respondents may keep multiple HITs open longer than necessary to avoid being penalized for responding too fast. However, it also could be that the MTurk survey showed respondents 1 item per page and required them to click “Next” to move to the next item, which can take longer [68]. If they chose to not answer an item, it also required that they validate their choice before moving forward. This same format was used for many of the items in the KnowledgePanel survey. However, when there were series of items all with the same response categories, Ipsos used an “accordion” format that showed all items in a set on 1 page and revealed the response categories for each as an answer for the previous item was selected. This is a format familiar to KnowledgePanel respondents and may have allowed them to move more quickly through these items.

#### Quality of Point Estimates

When the goal of data collection is to determine point estimates, it should be noted that standard statistics (eg, CIs) require that all members of the population of interest have a known, nonzero probability of being assigned to a survey [3,14,15]. Because workers on MTurk and similar opt-in panels select themselves into that panel and then find the HIT on their own rather than being selected to join a panel based on a known sampling frame and being randomly assigned to a survey as is done in KnowledgePanel, there is uncertainty about the inferences that can be made from the data [3,14]. Of course, low response rates can also impair probability-based panels’ ability to meet this known, nonzero probability of selection requirement, especially if nonresponders cannot be shown to be missing at random [15].

The KnowledgePanel completion rate (4134/7224, 57.2%) was higher than MTurk’s (6750/13,608, 49.6%); however, both are within the range observed in a large meta-analysis of online surveys [69] and higher than that study’s average response rate (44%). Nevertheless, the difference in completion rates between KnowledgePanel and MTurk was not as large as might be expected given that KnowledgePanel members were assigned the survey and the MTurk workers had to find it themselves through a posting. KnowledgePanel also had a higher completion rate on the 6-month follow-up survey (1205/1531, 82%) than MTurk (1077/1972, 55% at 3 months and 854/1972, 43% at 6 months). Part of this difference was likely because eligible KnowledgePanel respondents were assigned the 6-month follow-up survey, whereas the MTurk workers, known to have a high turnover rate [70], had to respond to an email inviting them again.

Bias due to self-selection and nonresponse can be addressed through adjustments such as weighting [3]. However, there is no guarantee that weighting will be successful; bias will only be reduced if the proper weighting variables are used, and they are often unknown [3,57,71,72]. We used the same variables for weighting the MTurk data as were used for the KnowledgePanel data with the exception of KnowledgePanel subdividing their regions into metro and nonmetro areas. As shown elsewhere [14,57] and as expected given its random assignment of surveys from a probability-based panel, the KnowledgePanel unweighted point estimates were closer to national benchmarks than the unweighted estimates from MTurk. However, even though MTurk’s unweighted point estimates for demographics exhibited the usual mismatch with national averages (younger, better educated, and lower income [6,10,15,18,73]), we were able to weight the MTurk data to match national estimates with a total absolute imbalance close to 0 (0.01). This weighting also improved MTurk’s PROMIS T-scores, resulting in estimates closer to national averages than those observed in KnowledgePanel. Weighting the MTurk sample also reduced the proportion endorsing each condition and generally moved prevalence closer to what was observed in KnowledgePanel. However, the maximum weight required for weighting the MTurk data was 30, which is quite high. Educational level was by far the most difficult variable to weight, which may be due to the unusual relationship between educational level and other variables observed in our correlation analyses (more on this in the following section). Nevertheless, as with any weighting procedure, this weighting may or may not have truly improved all point estimates [57,71,72,74].

Even after weighting, more KnowledgePanel than MTurk respondents had household incomes of >US $100,000 per year. Because there are fewer individuals with incomes of >US $100,000 than there are households with incomes of >US $100,000 [75], we checked the household size in each sample. The proportion of MTurk respondents reporting single-person households was lower than that for KnowledgePanel (880/5775, 15% vs 692/4115, 17%), making household size an unlikely explanation for the difference observed in household income levels.

#### Comparisons of Multivariate Analyses

The results of multivariate analyses might be more similar across platforms than univariate results [14,57,61-63]. In this study, we estimated and compared correlation coefficients for each platform. Overall, the correlation pairs demonstrated many similarities but also some important differences. Of the 105 correlation pairs between PROMIS measure variables, the mean absolute difference was 0.05, and the mean absolute difference between the additional 31 coefficients involving age and income was 0.07. However, the 17 correlation coefficient pairs involving educational level were dramatically different by platform— more than half had opposite signs (with MTurk correlations being the opposite from what would be expected from theory). In addition, the absolute difference among 7 correlation coefficient pairs was >0.20, and the largest difference was 0.29. These results are indicators of systematic differences between the 2 samples at least in terms of the relationship between educational level and PROMIS measures. Educational level also stood out as a problematic predictor of beliefs, attitudes, and knowledge in another study using Qualtrics Panels (a nonprobability panel selected using a variety of opt-in methods), suggesting that educational level might function differently in nonprobability panels than in nationally representative samples [15].

Similar to what was found in another study [76], 85% (41/48) of all the correlation coefficients in the full sample between PROMIS measures and demographics were larger in absolute value in MTurk than KnowledgePanel, suggesting an increased risk of false positives in those relationships. Slightly more (58/105, 55%) KnowledgePanel coefficients were larger than in MTurk when only considering correlations between PROMIS measures.

We only examined correlations between the variables we measured. Other researchers have suggested that the results of multivariate models, where the effects of several variables can be estimated at once, might differ less between probability and convenience samples, especially if the dependent variable is more “concrete” [15,77]. It has also been suggested that randomization to experimental conditions might also minimize biases observed in MTurk samples and offer internal validity at least as good as that observed in undergraduate student pools [15].

### Strengths and Limitations

This study benefited from large sample sizes and the opportunity to field essentially the same survey on 2 very different (convenience vs probability-based panel) platforms. However, there are some limitations.

First, the MTurk data were gathered earlier in the COVID-19 pandemic (August 31, 2021, to November 2, 2021) than the KnowledgePanel data (September 22, 2022, to October 2, 2022). The stage of the pandemic during which we gathered our data could have had an impact. However, as our health questions were about back pain and not about infectious disease, the main impact would likely be due to the inability to exercise or visit providers, especially earlier in the pandemic. This may have contributed to the slightly higher physical function and slightly lower pain interference T-scores in the KnowledgePanel data than in the MTurk data. However, other studies have also found worse health in nonprobability samples [72], and it is unclear whether this was a significant effect or whether these were the only effects of our timing.

Second, the KnowledgePanel sample included some respondents (5% [17]) who were provided with a device or internet connection to enable their participation, whereas everyone in the MTurk sample was required to have both [14,57].

Third, we only fielded the surveys in English.

Fourth, it is not likely that the use of the same fake conditions (“syndomitis” and “chekalism”) will continue to be as effective for prescreening a population as information about their use could spread among future respondents. This is similar to what happened over time to the use of “Bindro” as a fake drug name in surveys [78]. It is also possible that detecting careless respondents will be more challenging in the future as more sophisticated bots are used.

Fifth, other quality steps regarding the selection of workers on the MTurk platform may have resulted in different outcomes. For example, we chose to use workers with a good reputation (ie, those who had completed >500 HITs with a >95% approval rating). However, this may have limited us to the savvier survey takers. Other researchers have recommended recruiting naïve workers [27], and some studies are now recommending using those with a 99% approval rating [79] or using workers in CloudResearch’s Approved Participants group to improve MTurk data quality [80].

Sixth, a short initial prescreening survey could also be used to restrict an MTurk sample to better represent a segment of the US population (eg, younger adults [15]), or using CloudResearch to target workers may have resulted in a more nationally representative sample even though these respondents have been found to be less attentive and provide less reliable answers [18]. Nevertheless, MTurk and other nonprobability panels have also been found to be sufficiently accurate for certain types of studies. For example, a nonprobability sample should be sufficient if a researcher is investigating a phenomenon believed to be universal (ie, everyone would behave similarly) or one believed to be appropriately distributed in any large population so that the specific makeup of the sample is unlikely to affect conclusions [14,81].

### Conclusions

Similar to what has been shown in other studies [57,71], we found that a nationally representative probability-based sample resulted in higher-quality, more representative estimates compared to a convenience/nonprobability sample. Therefore, if cost is not a consideration, obtaining data from a nationally representative probability-based sample is recommended. However, given its substantially lower costs, interest in using platforms such as MTurk is likely to continue, and data quality was not so far from that observed for KnowledgePanel in our study as to negate any use of these convenience panels [15]. There are cases in which lower quality would be acceptable. For example, even if researchers can afford a probability-based panel, MTurk may be appropriate to generate hypotheses and pilot studies before preregistration and fielding [22].

Although the likelihood that a random sample is representative of a population could be determined if certain conditions are met, the accuracy of a nonprobability sample is nearly impossible to completely assess [14,15]. Nevertheless, a number of steps can improve the data collected from platforms such as MTurk. These improvements include making the survey available only once to high-quality (≥500 HITs and ≥95% approval rating) local (US IP addresses) workers in microbatches across the day and week, using a prescreening step (paid initial survey) that masks the existence of and eligibility criteria for any following more targeted surveys and includes fake or bogus items to identify those who might be misrepresenting themselves to qualify for further work, and weighting to match national benchmarks.

With appropriate prescreening and weighting, nonprobability samples can often be used but always with caution and awareness of methods to mitigate their shortcomings [15].

## Appendix

Multimedia Appendix 1 Item pairs used for consistency checks.

Multimedia Appendix 2 Correlations between various Patient-Reported Outcomes Measurement Information System scores and age, educational level, and income.

## Abbreviations

HIT: human intelligence task
MTurk: Mechanical Turk
PROMIS: Patient-Reported Outcomes Measurement Information System
PROMIS-29: 29-item Patient-Reported Outcomes Measurement Information System

## Data Availability

The datasets generated during and/or analyzed during this study are available in the Inter-university Consortium for Political and Social Research repository (OPENICPSR-198049) [47].

## References

[1] Chang L, Krosnick JA (2009). National surveys via RDD telephone interviewing versus the internet: comparing sample representativeness and response quality. Public Opin Q.

[2] Wyatt JC (2000). When to use web-based surveys. J Am Med Inform Assoc.

[3] Callegaro M, Baker R, Bethlehem J, Göritz AS, Krosnick JA, Lavrakas PJ, Callegaro M, Baker R, Bethlehem J, Goritz AS, Krosnick JA, Lavrakas PJ (2014). Online panel research: history, concepts, applications and a look at the future. Online Panel Research: A Data Quality Perspective.

[4] Trochim WM (2001). The Research Methods Knowledge Base.

[5] Chandler JJ, Paolacci G (2017). Lie for a Dime: when most prescreening responses are honest but most study participants are impostors. Soc Psychol Personal Sci.

[6] Paolacci G, Chandler J, Ipeirotis PG (2023). Running experiments on Amazon Mechanical Turk. Judgm Decis Mak.

[7] Thomas KA, Clifford S (2017). Validity and mechanical turk: an assessment of exclusion methods and interactive experiments. Comput Human Behav.

[8] Agley J, Xiao Y, Nolan R, Golzarri-Arroyo L (2022). Quality control questions on Amazon's Mechanical Turk (MTurk): a randomized trial of impact on the USAUDIT, PHQ-9, and GAD-7. Behav Res Methods.

[9] Peer E, Rothschild D, Gordon A, Evernden Z, Damer E (2022). Data quality of platforms and panels for online behavioral research. Behav Res Methods.

[10] Hitlin P (2016). Research in the crowdsourcing age: a case study. Pew Research Center.

[11] Sharpe Wessling K, Huber J, Netzer O (2017). MTurk character misrepresentation: assessment and solutions. J Consum Res.

[12] Wood D, Harms PD, Lowman GH, DeSimone JA (2017). Response speed and response consistency as mutually validating indicators of data quality in online samples. Soc Psychol Personal Sci.

[13] MacInnis CC, Boss HC, Bourdage JS (2020). More evidence of participant misrepresentation on Mturk and investigating who misrepresents. Pers Individ Dif.

[14] Cornesse C, Blom AG, Dutwin D, Krosnick JA, De Leeuw ED, Legleye S, Pasek J, Pennay D, Phillips B, Sakshaug JW, Struminskaya B, Wenz A (2020). A review of conceptual approaches and empirical evidence on probability and nonprobability sample survey research. J Surv Stat Methodol.

[15] Zack ES, Kennedy J, Long JS (2019). Can nonprobability samples be used for social science research? A cautionary tale. Surv Res Methods.

[16] Hays RD, Liu H, Kapteyn A (2015). Use of Internet panels to conduct surveys. Behav Res Methods.

[17] Bradley VC, Kuriwaki S, Isakov M, Sejdinovic D, Meng XL, Flaxman S (2021). Unrepresentative big surveys significantly overestimated US vaccine uptake. Nature.

[18] Chandler J, Rosenzweig C, Moss AJ, Robinson J, Litman L (2019). Online panels in social science research: expanding sampling methods beyond Mechanical Turk. Behav Res Methods.

[19] Mortensen K, Hughes TL (2018). Comparing Amazon's Mechanical Turk Platform to conventional data collection methods in the health and medical research literature. J Gen Intern Med.

[20] von Elm E, Altman DG, Egger M, Pocock SJ, Gøtzsche PC, Vandenbroucke JP (2007). The strengthening the Reporting of Observational Studies in Epidemiology (STROBE) statement: guidelines for reporting observational studies. Lancet.

[21] Eysenbach G (2004). Improving the quality of web surveys: the Checklist for Reporting Results of Internet E-Surveys (CHERRIES). J Med Internet Res.

[22] Kennedy R, Clifford S, Burleigh T, Waggoner PD, Jewell R, Winter NJ (2020). The shape of and solutions to the MTurk quality crisis. Political Sci Res Methods.

[23] Mason W, Suri S (2012). Conducting behavioral research on Amazon's Mechanical Turk. Behav Res Methods.

[24] Ipeirotis PG (2010). Analyzing the Amazon Mechanical Turk marketplace. The ACM Magazine for Students.

[25] Kim HS, Hodgins DC (2020). Are you for real? Maximizing participant eligibility on Amazon's Mechanical Turk. Addiction.

[26] Hydock C (2018). Assessing and overcoming participant dishonesty in online data collection. Behav Res Methods.

[27] Robinson J, Rosenzweig C, Moss AJ, Litman L (2019). Tapped out or barely tapped? Recommendations for how to harness the vast and largely unused potential of the Mechanical Turk participant pool. PLoS One.

[28] How to make $50 a day on MTurk: the ultimate guide in 2023. Finance Over Fifty.

[29] (2023). Definitive guide on how to make money on Amazon MTurk in 2024. Gigworker.

[30] FAQs. Amazon Mechanical Turk Worker.

[31] Bertoni N KnowledgePanel. Ipsos.

[32] Crowther M, Thomas N (2019). Ipsos acquires GfK's KnowledgePanel. Daily Research News Online.

[33] Kaufman DJ, Baker R, Milner LC, Devaney S, Hudson KL (2016). A survey of U.S adults' opinions about conduct of a nationwide Precision Medicine Initiative® cohort study of genes and environment. PLoS One.

[34] (2023). KnowledgePanel®: a methodological overview. Ipsos.

[35] Hays RD, Qureshi N, Herman PM, Rodriguez A, Kapteyn A, Edelen MO (2023). Effects of excluding those who report having "syndomitis" or "chekalism" on data quality: longitudinal health survey of a sample from Amazon's Mechanical Turk. J Med Internet Res.

[36] Goodman JK, Cryder CE, Cheema A (2012). Data collection in a flat world: the strengths and weaknesses of Mechanical Turk samples. J Behav Decis Mak.

[37] Peer E, Vosgerau J, Acquisti A (2014). Reputation as a sufficient condition for data quality on Amazon Mechanical Turk. Behav Res Methods.

[38] Herman PM, Edelen MO, Rodriguez A, Hilton LG, Hays RD (2020). A protocol for chronic pain outcome measurement enhancement by linking PROMIS-29 scale to legacy measures and improving chronic pain stratification. BMC Musculoskelet Disord.

[39] Chmielewski M, Kucker SC (2019). An MTurk crisis? Shifts in data quality and the impact on study results. Soc Psychol Personal Sci.

[40] Walters K, Christakis DA, Wright DR (2018). Are Mechanical Turk worker samples representative of health status and health behaviors in the U.S.?. PLoS One.

[41] Litman L, Robinson J, Abberbock T (2017). TurkPrime.com: a versatile crowdsourcing data acquisition platform for the behavioral sciences. Behav Res Methods.

[42] Meade AW, Craig SB (2012). Identifying careless responses in survey data. Psychol Methods.

[43] mturk forum. XenForo™.

[44] Welcome to MTurk Crowd. MTurk Crowd.

[45] The Mechanical Turk (mTurk) hub. mTurk Forum by TurkerView.

[46] r/HITsWorthTurkingFor. Reddit.

[47] Find data. Institute for Social Research (ICPSR).

[48] Cohen J (1960). A coefficient of agreement for nominal scales. Educ Psychol Meas.

[49] Landis JR, Koch GG (1977). The measurement of observer agreement for categorical data. Biometrics.

[50] McHugh ML (2012). Interrater reliability: the kappa statistic. Biochem Med.

[51] Kim Y, Dykema J, Stevenson J, Black P, Moberg DP (2018). Straightlining: overview of measurement, comparison of indicators, and effects in mail–web mixed-mode surveys. Soc Sci Comput Rev.

[52] Cronbach LJ (1951). Coefficient alpha and the internal structure of tests. Psychometrika.

[53] Shapiro DN, Chandler J, Mueller PA (2013). Using Mechanical Turk to study clinical populations. Clin Psychol Sci.

[54] Feldt LS, Woodruff DJ, Salih FA (1987). Statistical inference for coefficient alpha. Appl Psychol Meas.

[55] Diedenhofen B, Musch J (2016). cocron: a web interface and R package for the statistical comparison of Cronbach’s Alpha coefficients. Int J Inf Sci.

[56] Wilcox R, Wilcox R (2012). A foundation for robust methods. Introduction to Robust Estimation and Hypothesis Testing: A volume in Statistical Modeling and Decision Science.

[57] Callegaro M, Villar A, Yeager D, Krosnick JA, Callegaro M, Baker R, Bethlehem J, Göritz AS, Krosnick JA, Lavrakas PJ (2014). A critical review of studies investigating the quality of data obtained with online panels based on probability and nonprobability samples. Online Panel Research.

[58] Cella D, Choi SW, Condon DM, Schalet B, Hays RD, Rothrock NE, Yount S, Cook KF, Gershon RC, Amtmann D, DeWalt DA, Pilkonis PA, Stone AA, Weinfurt K, Reeve BB (2019). PROMIS adult health profiles: efficient short-form measures of seven health domains. Value Health.

[59] Hays RD, Spritzer KL, Schalet BD, Cella D (2018). PROMIS-29 v2.0 profile physical and mental health summary scores. Qual Life Res.

[60] Goel S, Obeng A, Rothschild D (2015). Non-representative surveys: fast, cheap, and mostly accurate. Work Pap.

[61] Berrens RP, Bohara AK, Jenkins-Smith H, Silva C, Weimer DL (2017). The advent of internet surveys for political research: a comparison of telephone and internet samples. Polit Anal.

[62] Pasek J (2015). When will nonprobability surveys mirror probability surveys? Considering types of inference and weighting strategies as criteria for correspondence. Int J Public Opin Res.

[63] Groves RM (2004). Survey Errors and Survey Costs.

[64] Hays RD, Bjorner JB, Revicki DA, Spritzer KL, Cella D (2009). Development of physical and mental health summary scores from the Patient-Reported Outcomes Measurement Information System (PROMIS) global items. Qual Life Res.

[65] Deyo RA, Dworkin SF, Amtmann D, Andersson G, Borenstein D, Carragee E, Carrino J, Chou R, Cook K, DeLitto A, Goertz C, Khalsa P, Loeser J, Mackey S, Panagis J, Rainville J, Tosteson T, Turk D, Von Korff M, Weiner DK (2014). Report of the NIH Task Force on research standards for chronic low back pain. Pain Med.

[66] Morbidity and mortality weekly report (MMWR): national, state-level, and county-level prevalence estimates of adults aged ≥18 years self-reporting a lifetime diagnosis of depression — United States, 2020. Centers for Disease Control and Prevention.

[67] Zajacova A, Lawrence EM (2018). The relationship between education and health: reducing disparities through a contextual approach. Annu Rev Public Health.

[68] Hays RD, Bode R, Rothrock N, Riley W, Cella D, Gershon R (2010). The impact of next and back buttons on time to complete and measurement reliability in computer-based surveys. Qual Life Res.

[69] Wu MJ, Zhao K, Fils-Aime F (2022). Response rates of online surveys in published research: a meta-analysis. Comput Hum Behav Rep.

[70] Stewart N, Ungemach C, Harris AJ, Bartels DM, Newell BR, Paolacci G, Chandler J (2023). The average laboratory samples a population of 7,300 Amazon Mechanical Turk workers. Judgm Decis Mak.

[71] Yeager DS, Krosnick JA, Chang L, Javitz HS, Levendusky MS, Simpser A, Wang R (2011). Comparing the accuracy of RDD telephone surveys and internet surveys conducted with probability and non-probability samples. Public Opin Q.

[72] Schnell R, Klingwort J (2024). Health estimate differences between six independent web surveys: different web surveys, different results?. BMC Med Res Methodol.

[73] Qureshi N, Edelen M, Hilton L, Rodriguez A, Hays RD, Herman PM (2022). Comparing data collected on Amazon's Mechanical Turk to national surveys. Am J Health Behav.

[74] MacInnis B, Krosnick JA, Ho AS, Cho MJ (2018). The accuracy of measurements with probability and nonprobability survey samples: replication and extension. Public Opin Q.

[75] Current population survey annual social and economic supplement. US Census Bureau.

[76] Pasek J, Krosnick JA Measuring intent to participate and participation in the 2010 census and their correlates and trends: comparisons of RDD telephone and non-probability sample internet survey data. United States Census Bureau.

[77] Simmons AD, Bobo LD (2015). Can non-full-probability internet surveys yield useful data? A comparison with full-probability face-to-face surveys in the domain of race and social inequality attitudes. Sociol Methodol.

[78] (2005). Bindro by Corbyn. Urban Dictionary.

[79] Matherly T (2019). A panel for lemons? Positivity bias, reputation systems and data quality on MTurk. Eur J Mark.

[80] Hauser DJ, Moss AJ, Rosenzweig C, Jaffe SN, Robinson J, Litman L (2023). Evaluating CloudResearch's approved group as a solution for problematic data quality on MTurk. Behav Res Methods.

[81] Coppock A, Leeper TJ, Mullinix KJ (2018). Generalizability of heterogeneous treatment effect estimates across samples. Proc Natl Acad Sci U S A.

