# Supplementary material for: Comparing Health Survey Data Cost and Quality Between Amazon’s Mechanical Turk and Ipsos’ KnowledgePanel: Observational Study
Source: J Med Internet Res. 2024 Nov 29;26:e63032. doi: 10.2196/63032 (PMC11645511; doi:10.2196/63032)
Supplement: Multimedia Appendix 1 [file jmir_v26i1e63032_app1.docx]

Appendix A. Pairs of items used for consistency checks

2 Pain interference items

- What number best describes how, during the past week, pain has interfered with your general activity? (Scale: 0-10)
- In the past 6 months, how much has back pain interfered with your daily activities rated on a 0-10 scale where 0 is "no interference" and 10 is "unable to carry on any activities"? (Scale: 0-10)

2 Trouble doing items

- I have trouble doing all of my regular leisure activities with others (Scale: Never to always)
- I have trouble participating in recreational activities with others (Scale: Never to always)

2 Problems with sleep items

- I had a problem with my sleep (Scale: Not at all to Very Much)
- I had difficulty falling asleep (Scale: Not at all to Very Much)

2 Able to concentrate/focus items

- I have been able to concentrate (Scale: Not at all to Very Much)
- I have been able to focus my attention (Scale: Not at all to Very Much)

2 Memory items

- I have been able to remember to do things, like take medicine or buy something I need (Scale: Not at all to Very Much)
- My memory has been as good as usual (Scale: Not at all to Very Much)
