# Supplementary material for: Comparing Health Survey Data Cost and Quality Between Amazon’s Mechanical Turk and Ipsos’ KnowledgePanel: Observational Study
Source: J Med Internet Res. 2024 Nov 29;26:e63032. doi: 10.2196/63032 (PMC11645511; doi:10.2196/63032)
Supplement: Multimedia Appendix 2 [file jmir_v26i1e63032_app2.docx]

Appendix B. Full sample: correlations between various PROMIS scores and age, education and income: MTurk correlations above, KnowledgePanel correlations below

|  | Physical Function | Anxiety | Depression | Fatigue | Sleep | Social Roles | Pain Interference | Pain Intensity |
| --- | --- | --- | --- | --- | --- | --- | --- | --- |
| Anxiety | -0.43 (-0.45, -0.41) -0.30 (-0.32, -0.27) |  |  |  |  |  |  |  |
| Depression | -0.43 (-0.45, -0.41) -0.34 (-0.36, -0.31) | 0.82 (0.81, 0.83) 0.83 (0.82, 0.84) |  |  |  |  |  |  |
| Fatigue | -0.47 (-0.49, -0.45) -0.49 (-0.51, -0.47) | 0.70 (0.69, 0.71) 0.60 (0.58, 0.62) | 0.71 (0.69, 0.72) 0.60 (0.58, 0.62) |  |  |  |  |  |
| Sleep | -0.30 (-0.32, -0.27) -0.35 (-0.38, -0.33) | 0.52 (0.51, 0.54) 0.50 (0.48, 0.52) | 0.53 (0.51, 0.55) 0.50 (0.48, 0.53) | 0.61 (0.59, 0.63) 0.59 (0.57, 0.61) |  |  |  |  |
| Social Roles | 0.64 (0.62, 0.65) 0.66 (0.64, 0.68) | -0.66 (-0.68, -0.65) -0.56 (-0.58, -0.54) | -0.66 (-0.68, -0.65) -0.59 (-0.60, -0.56) | -0.68 (-0.69, -0.67) -0.68 (-0.69, -0.66) | -0.49 (-0.51, -0.47) -0.52 (-0.54, -0.50) |  |  |  |
| Pain Interference | -0.71 (-0.73, -0.70) -0.71 (-0.72, -0.69) | 0.51 (0.49, 0.53) 0.37 (0.35, 0.40) | 0.50 (0.48, 0.52) 0.40 (0.37, 0.42) | 0.54 (0.52, 0.56) 0.55 (0.52, 0.57) | 0.37 (0.34, 0.39) 0.44 (0.41, 0.46) | -0.71 (-0.73, -0.70) -0.66 (-0.68, -0.64) |  |  |
| Pain Intensity | -0.59 (-0.61, -0.58) -0.58 (-0.60, -0.56) | 0.46 (0.44, 0.48) 0.32 (0.30, 0.35) | 0.45 (0.43, 0.47) 0.34 (0.32, 0.37) | 0.48 (0.46, 0.50) 0.48 (0.46, 0.51) | 0.37 (0.35, 0.39) 0.42 (0.39, 0.44) | -0.56 (-0.57, -0.54) -0.53 (-0.55, -0.50) | 0.72 (0.71, 0.73) 0.76 (0.74, 0.77) |  |
| Cognitive Function | 0.33 (0.30, 0.35) 0.24 (0.21, 0.27) | -0.37 (-0.39, -0.34) -0.37 (-0.39, -0.34) | -0.37 (-0.40, -0.35) -0.37 (-0.40, -0.34) | -0.30 (-0.33, -0.28) -0.32 (-0.35, -0.29) | -0.31 (-0.33, -0.29) -0.35 (-0.38, -0.32) | 0.39 (0.37, 0.41) 0.36 (0.33, 0.38) | -0.31 (-0.33, -0.29) -0.26 (-0.29, -0.23) | -0.29 (-0.31, -0.27) -0.23 (-0.26, -0.20) |
| PROMIS-29 Mental Health Summary | 0.59 (0.58, 0.61) 0.60 (0.58, 0.62) | -0.83 (-0.84, -0.83) -0.76 (-0.77, -0.75) | -0.84 (-0.84, -0.83) -0.77 (-0.78, -0.76) | -0.91 (-0.92, -0.91) -0.91 (-0.91, -0.90) | -0.70 (-0.71, -0.69) -0.72 (-0.73, -0.70) | 0.86 (0.85, 0.87) 0.85 (0.84, 0.86) | -0.71 (-0.72, -0.70) -0.70 (-0.71, -0.68) | -0.64 (-0.65, -0.62) -0.62 (-0.64, -0.60) |
| PROMIS-29 Physical Health Summary | 0.99 (0.99, 0.99) 0.99 (0.99, 0.99) | -0.50 (-0.52, -0.48) -0.35 (-0.38, -0.33) | -0.50 (-0.52, -0.48) -0.39 (-0.42, -0.37) | -0.54 (-0.55, -0.52) -0.55 (-0.57, -0.53) | -0.35 (-0.37, -0.33) -0.41 (-0.43, -0.38) | 0.73 (0.71, 0.74) 0.74 (0.72, 0.75) | -0.78 (-0.79, -0.77) -0.77 (-0.78, -0.76) | -0.66 (-0.68, -0.65) -0.65 (-0.66, -0.63) |
| PROPr utility | 0.69 (0.67, 0.70) 0.69 (0.67, 0.71) | -0.71 (-0.72, -0.70) -0.61 (-0.62, -0.59) | -0.75 (-0.77, -0.74) -0.65 (-0.67, -0.64) | -0.75 (-0.76, -0.74) -0.73 (-0.75, -0.72) | -0.64 (-0.66, -0.63) -0.66 (-0.68, -0.64) | 0.79 (0.78, 0.80) 0.78 (0.77, 0.79) | -0.71 (-0.72, -0.70) -0.70 (-0.71, -0.68) | -0.61 (-0.62, -0.59) -0.58 (-0.60, -0.56) |
| Global Physical | 0.64 (0.62, 0.65) 0.70 (0.69, 0.72) | -0.54 (-0.56, -0.52) -0.46 (-0.48, -0.43) | -0.55 (-0.57, -0.53) -0.49 (-0.51, -0.47) | -0.66 (-0.68, -0.65) -0.68 (-0.70, -0.67) | -0.53 (-0.55, -0.51) -0.54 (-0.56, -0.52) | 0.63 (0.61, 0.64) 0.68 (0.67, 0.70) | -0.63 (-0.65, -0.62) -0.70 (-0.72, -0.69) | -0.72 (-0.73, -0.71) -0.75 (-0.76, -0.73) |
| Global Mental | 0.16 (0.14, 0.19) 0.34 (0.31, 0.36) | -0.54 (-0.56, -0.52) -0.65 (-0.67, -0.63) | -0.58 (-0.59, -0.56) -0.68 (-0.69, -0.66) | -0.53 (-0.55, -0.51) -0.56 (-0.58, -0.54) | -0.53 (-0.55, -0.51) -0.54 (-0.56, -0.52) | 0.38 (0.35, 0.40) 0.54 (0.52, 0.56) | -0.18 (-0.20, -0.15) -0.37 (-0.40, -0.34) | -0.23 (-0.25, -0.20) -0.35 (-0.38, -0.32) |
| ISS Score | -0.86 (-0.87, -0.85) -0.86 (-0.86, -0.85) | 0.52 (0.50, 0.54) 0.37 (0.35, 0.40) | 0.52 (0.50, 0.54) 0.41 (0.39, 0.44) | 0.56 (0.55, 0.58) 0.56 (0.54, 0.58) | 0.38 (0.35, 0.40) 0.44 (0.42, 0.47) | -0.73 (-0.74, -0.71) -0.71 (-0.72, -0.69) | 0.91 (0.90, 0.91) 0.90 (0.90, 0.91) | 0.82 (0.81, 0.83) 0.83 (0.82, 0.84) |
| Age | -0.09 (-0.12, -0.06) -0.27 (-0.30, -0.24) | -0.15 (-0.18, -0.13) -0.22 (-0.25, -0.19) | -0.16 (-0.19, -0.13) -0.19 (-0.22, -0.16) | -0.08 (-0.11, -0.06) -0.10 (-0.13, -0.07) | -0.05 (-0.08, -0.03) -0.11 (-0.14, -0.08) | 0.05 (0.02, 0.07) -0.05 (-0.08, -0.02) | 0.03 (0.00, 0.05) 0.15 (0.12, 0.18) | 0.02 (-0.01, 0.04) 0.12 (0.09, 0.15) |
| Education | -0.07 (-0.09, -0.04) 0.19 (0.16, 0.22)* | 0.02 (-0.01, 0.04) -0.08 (-0.11, -0.05) | -0.02 (-0.04, 0.01) -0.12 (-0.15, -0.09) | -0.03 (-0.06, -0.01) -0.05 (-0.08, -0.02) | -0.10 (-0.12, -0.07) -0.09 (-0.13, -0.06) | -0.04 (-0.07, -0.01) 0.12 (0.09, 0.15) | 0.09 (0.06, 0.12)* -0.16 (-0.19, -0.13) | 0.06 (0.04, 0.09)* -0.18 (-0.21, -0.15) |
| Income | 0.17 (0.15, 0.20) 0.26 (0.23, 0.29) | -0.14 (-0.17, -0.12) -0.17 (-0.20, -0.14) | -0.17 (-0.20, -0.15) -0.19 (-0.22, -0.16) | -0.11 (-0.14, -0.09) -0.13 (-0.16, -0.10) | -0.12 (-0.15, -0.10) -0.13 (-0.16, -0.10) | 0.14 (0.12, 0.17) 0.21 (0.18, 0.24) | -0.14 (-0.16, -0.11) -0.24 (-0.27, -0.22) | -0.10 (-0.13, -0.08) -0.24 (-0.27, -0.21) |

|  | Cognitive Function | PROMIS-29 Mental Health Summary | PROMIS-29 Physical Health Summary | PROPr utility | Global Physicial | Mental | ISS Score | Age | Education |
| --- | --- | --- | --- | --- | --- | --- | --- | --- | --- |
| PROMIS-29 Mental Health Summary | 0.41 (0.38, 0.43) 0.41 (0.38, 0.43) |  |  |  |  |  |  |  |  |
| PROMIS-29 Physical Health Summary | 0.36 (0.33, 0.38) 0.27 (0.24, 0.30) | 0.68 (0.67, 0.69) 0.68 (0.66, 0.70) |  |  |  |  |  |  |  |
| PROPr utility | 0.68 (0.67, 0.70) 0.68 (0.66, 0.70) | 0.88 (0.87, 0.89) 0.86 (0.86, 0.87) | 0.75 (0.74, 0.76) 0.75 (0.73, 0.76) |  |  |  |  |  |  |
| Global Physicial | 0.35 (0.33, 0.37) 0.35 (0.32, 0.38) | 0.74 (0.73, 0.75) 0.77 (0.76, 0.79) | 0.69 (0.67, 0.70) 0.75 (0.74, 0.77) | 0.71 (0.70, 0.72) 0.74 (0.73, 0.76) |  |  |  |  |  |
| Mental | 0.23 (0.20, 0.25) 0.38 (0.35, 0.41) | 0.56 (0.54, 0.58) 0.68 (0.66, 0.70) | 0.21 (0.18, 0.23) 0.39 (0.36, 0.41) | 0.46 (0.44, 0.48) 0.60 (0.58, 0.62) | 0.55 (0.53, 0.57) 0.62 (0.60, 0.64) |  |  |  |  |
| ISS Score | -0.32 (-0.35, -0.30) -0.28 (-0.31, -0.26) | -0.73 (-0.74, -0.71) -0.71 (-0.73, -0.70) | -0.90 (-0.91, -0.90) -0.90 (-0.90, -0.89) | -0.74 (-0.75, -0.73) -0.73 (-0.75, -0.72) | -0.71 (-0.72, -0.70) -0.79 (-0.80, -0.78) | -0.19 (-0.22, -0.17) -0.39 (-0.42, -0.37)* |  |  |  |
| Age | 0.18 (0.16, 0.21) 0.17 (0.14, 0.20) | 0.09 (0.07, 0.12) 0.08 (0.05, 0.11) | -0.07 (-0.10, -0.05) -0.25 (-0.27, -0.22) | 0.09 (0.06, 0.12) 0.03 (-0.00, 0.06) | -0.01 (-0.03, 0.02) -0.09 (-0.12, -0.06) | 0.08 (0.06, 0.11) 0.20 (0.17, 0.23) | 0.05 (0.03, 0.08) 0.18 (0.15, 0.21) |  |  |
| Education | -0.03 (-0.06, -0.01) 0.16 (0.13, 0.19) | 0.01 (-0.02, 0.03) 0.12 (0.09, 0.15) | -0.07 (-0.10, -0.04) 0.19 (0.16, 0.22)* | -0.02 (-0.05, 0.00) 0.18 (0.15, 0.21)* | 0.05 (0.02, 0.08) 0.21 (0.18, 0.24) | 0.20 (0.17, 0.22) 0.18 (0.15, 0.21) | 0.08 (0.05, 0.10)* -0.21 (-0.24, -0.18) | 0.01 (-0.02, 0.04) 0.06 (0.02, 0.09) |  |
| Income | 0.13 (0.10, 0.15) 0.17 (0.14, 0.20) | 0.16 (0.13, 0.18) 0.21 (0.18, 0.24) | 0.18 (0.15, 0.20) 0.27 (0.24, 0.30) | 0.18 (0.16, 0.21) 0.25 (0.22, 0.28) | 0.18 (0.16, 0.21) 0.27 (0.25, 0.30) | 0.21 (0.18, 0.23) 0.25 (0.22, 0.28) | -0.14 (-0.17, -0.12) -0.28 (-0.31, -0.26) | 0.02 (-0.01, 0.04) -0.02 (-0.05, 0.01) | 0.27 (0.25, 0.29)* 0.45 (0.42, 0.47) |

Underline indicates absolute value differences in correlations of >0.1. * = correlation differences >.2.

Grey indicates correlations going in the opposite direction.
